# Unconscious mind activates central cardiovascular network and promotes adaptation to microgravity possibly anti-aging during 1-year-long spaceflight

**DOI:** 10.1038/s41598-022-14858-8

**Published:** 2022-07-13

**Authors:** Kuniaki Otsuka, Germaine Cornelissen, Satoshi Furukawa, Koichi Shibata, Yutaka Kubo, Koh Mizuno, Tatsuya Aiba, Hiroshi Ohshima, Chiaki Mukai

**Affiliations:** 1 Executive Medical Center, Related Medical Facility, Totsuka Royal Clinic, Tokyo Women’s Medical University, Sinjuku City, Tokyo, Japan; 2 Halberg Chronobiology Center, University of Minnesota, Minneapolis, MN USA; 3 Space Biomedical Research Group, Japan Aerospace Exploration Agency, Ibaraki, Japan; 4 Department of Medicine, Medical Center East, Tokyo Women’s Medical University, Tokyo, Japan; 5 Faculty of Education, Tohoku Fukushi University, Miyagi, Japan; 6 Tokyo University of Science, Tokyo, Japan

**Keywords:** Computational biology and bioinformatics, Evolution, Neuroscience, Physiology, Systems biology, Planetary science, Cardiology, Health care, Medical research, Neurology, Astronomy and planetary science

## Abstract

The intrinsic cardiovascular regulatory system (β, 0.00013–0.02 Hz) did not adapt to microgravity after a 6-month spaceflight. The infraslow oscillation (ISO, 0.01–0.10 Hz) coordinating brain dynamics via thalamic astrocytes plays a key role in the adaptation to novel environments. We investigate the adaptive process of a healthy astronaut during a 12-month-long spaceflight by analyzing heart rate variability (HRV) in the LF (0.01–0.05 Hz) and MF1 (0.05–0.10 Hz) bands for two consecutive days on four occasions: before launch, at 1-month (ISS01) and 11-month (ISS02) in space, and after return to Earth. Alteration of β during ISS01 improved during ISS02 (*P* = 0.0167). During ISS01, LF and MF1 bands, reflecting default mode network (DMN) activity, started to increase at night (by 43.1% and 32.0%, respectively), when suprachiasmatic astrocytes are most active, followed by a 25.9% increase in MF1-band throughout the entire day during ISS02, larger at night (47.4%) than during daytime. Magnetic declination correlated positively with β during ISS01 (r = 0.6706, P < 0.0001) and ISS02 (r = 0.3958, *P* = 0.0095). Magnetic fluctuations may affect suprachiasmatic astrocytes, and the DMN involving ISOs and thalamic astrocytes may then be activated, first at night, then during the entire day, a mechanism that could perhaps promote an anti-aging effect noted in other investigations.

## Introduction

Exposure to microgravity in space heavily influences human physiology, resulting in cardiovascular dysfunction, immune suppression and impaired secretion of hormones and neurotransmitters. Microgravity-induced blood volume redistribution is the initial trigger to cardiovascular dysfunction, involving diverse processes and complex mechanisms^1–3^. As we reported previously^4,5^, the “intrinsic” cardiovascular regulatory system (β)^6,7^ did not adapt to space microgravity even during a 6-month spaceflight. Astronauts living on the International Space Station (ISS) also experience other unique stressors, including cosmic radiation, noise, social isolation, and confinement, factors that can impact human aging. It was once widely accepted that the space environment accelerates the aging process^8,9^. Unexpectedly, however, recent research suggested that genes involved in *Caenorhabditis elegans* lifespan extension were up-regulated after spaceflight^10^ and that spaceflight could extend lifespan in *Drosophila*^11^. With the increasing duration of current and planned spaceflight missions, studying the effect of the space environment on lifespan has increased in interest and medical importance. Studies are needed to determine whether long-duration spaceflight affects human well-being and aging.

Several recent investigations suggest that long-duration space travel may be associated with anti-aging effects. In the National Aeronautics and Space Administration (NASA) Twin Study, the identical twin astronaut monitored before, during, and after a 1-year mission onboard the ISS had lengthened telomeres upon return to Earth as compared to his twin brother who served as a genetically matched ground control^12,13^. Because telomere length is considered a marker of cellular aging, aging being usually associated with decreased telomere length^14,15^, the NASA twin study suggests a possible anti-aging effect of long-duration space travel. Another study of blood DNA methylation in six participants of the Mars-500 mission, a high-fidelity 520-day ground simulation experiment, showed significant decreases in epigenetic aging at the 168- and 300-day time points^16^.

We also found in one study of seven astronauts that 6-month space flights led to improved heart rate variability (HRV), reflecting good health and anti-aging^17^. In another study of ten astronauts living on the ISS for 6 months, we found that the circadian rhythm of heart rate (HR) was strengthened in space, sleep quality was improved, and parasympathetic tone was enhanced at night, thereby perhaps conveying an anti-aging effect^18^. Surprisingly, we also found that magnetic fluctuations affect activity of the brain’s default mode network (DMN) by stimulating the VLF (0.003–0.04 Hz) component of HRV in a light-dependent manner and/or with help from the circadian clock. These findings suggest that the unconscious activation of the brain’s functional network took place in long-duration space travel, promoting well-being and perhaps anti-aging properties, as discussed previously^17–19^.

Human senses can handle more than 11 million bits/s: 10 million are processed by the visual system; about 1 million via the ears; 10,000 to 100,000 via the skin; much fewer by the olfactory system and other sensory channels (gustatory and vestibular). Only a minuscule proportion of sensory signals (about 50 bits/s), however, is processed by the conscious mind. The remaining signals are processed unconsciously, amounting to about 220,000 (11,000,000/50) times the signals processed consciously.

Multiple lines of converging evidence point to the infraslow oscillation (ISO, 0.01–0.10 Hz), approximately 0.02 Hz (1/min) fluctuations, as underlying the unconscious processing of information among resting state networks in humans^20–23^. Unconscious information processing has been described as flexible, sharing many sophisticated characteristics with its conscious counterpart, involving the dynamics of large-scale brain networks, including the lateral and medial *orbitofrontal cortex*, *prefrontal cortex*, *insula*, anterior *cingulate cortex*, *hippocampus*, *amygdala*, posterior *cingulate cortex*, *precuneus*, and *thalamus*^19,24,25^. Recent physiological investigations suggest that the ISO, which coordinates brain dynamics without conscious attention via thalamic astrocytes, plays a key role in the adaptation to novel environments, together with astrocytes in the suprachiasmatic nuclei (SCN), which are active during the night^26–28^.

For over 40 years, cardiovascular adaptations to microgravity in space have been investigated in humans^29–42^. However, as sample sizes were small and conditions of data collection were variable, results lack consistency. Measurements of HR and HRV in space showed individual differences and did not provide unequivocal results, sometimes showing decreases, increases, or no change compared to preflight values^5,17,18,38,43^. Hence, we must cautiously assess and understand the environment in which astronauts work, live and adapt in space, and for how long they stayed in space after launch.

Baevsky et al.^43^ first reported adaptive responses of two crewmembers during a 175-day space mission. They found that microgravity led to marked signs of activation of the sympathetic nervous system, based on an increase in HR and a decrease in HF power^43^. They identified three consecutive stages of adaptation, also in a cosmonaut studied during a 438-day mission on the MIR station. First, ultradian rhythms at night reflect activation of the subcortical cardiovascular centers and of the higher levels of autonomic regulation at 8 to 9 months after launch. Second, HR decreases in association with an increase in cardiac contractility during the last few months in space. Third, an increase in the absolute power of the LF component reflects involvement of the vasomotor center. Baevsky et al.^44^ postulated that the central levels of circulatory regulation were activated, as evidenced by the presence of about-90-min (ultradian) rhythms at night. Our own studies also indicate that the brain’s DMN was activated in space in a light-dependent manner and/or with help from the circadian clock^30,44,45^.

Since this pioneering study^44^, long-term spaceflights only lasted about 6 months until only recently. Herein, we had a chance to investigate the adaptive process of the autonomic system during a 12-month-long mission in space. In particular, we observed how the ISO mediates the adaptive process to promote well-being and perhaps anti-aging in a healthy astronaut.

## Subject and methods

### Subject

A clinically healthy astronaut participated in the ISS JAXA (Japan Aerospace Exploration Agency) investigation named “Biological Rhythms 48 Hrs; 12 months in ISS orbit”. The astronaut had passed class III physical examinations from NASA. The Institutional Review Boards of NASA, ESA (European Space Agency), JAXA and Human Research Multilateral Review Board approved the study. The astronaut provided written informed consent after receiving a detailed explanation of the study protocol, according to the Declaration of Helsinki Principles. All methods were performed in accordance with the JAXA/ESA/NASA guidelines and regulations.

### Experimental protocols

Ambulatory around-the-clock 48-h ECG records were obtained with a two-channel Holter recorder (FM-180; Fukuda Denshi, Tokyo, Japan or H12 + ; Mortara, NY, USA). Measurements were made four times: once, 156–157 days before launch (Before); twice during flight on the ISS: ISS01 (18–19 days), and ISS02 (326–327 days) after launch; and once, 103–104 days after return to Earth (After). Actigraphic recordings were obtained twice (Before and After) for about 4 days, using wrist-borne actiwatches (Actiwatch Spectrum; Phillips/Respironics, Murrysville, USA) on the non-dominant wrist, providing sleep/wake activity in 1-min epochs^46–48^.

### Analysis of heart rate variability and activity

Data collection and measurement procedures were conducted as previously reported^4,5,17–19,49^. Briefly, for HRV measurements, QRS waveforms were read from continuous ECG records. The RR intervals between normal QRS waveforms were extracted as normal-to-normal (NN) intervals, which were A/D converted (125-Hz) with 8-ms time resolution. After the authors confirmed that all artifacts were actually removed and that the data excluded supraventricular or ventricular arrhythmia, time-domain HRV indices (r-MSSD and pNN50), and conventional frequency-domain measures (HF: 0.15–0.40 Hz, LF: 0.04–0.15 Hz, and VLF: 0.003–0.04 Hz)^50^ were obtained with the MemCalc/CHIRAM (Suwa Trust GMS, Tokyo, Japan) software^51^. Time series of NN intervals were also processed consecutively in 180-min intervals, progressively displaced by 5 min, to estimate TF (0.0001–0.50 Hz), ULF (0.0001–0.003 Hz), and the 1/f^β^-type scaling in HRV. Focus was placed on the 0.00013–0.02 Hz frequency range (periods of 2.0 h to 0.83 min).

Time series of NN intervals covering 5-min intervals were processed consecutively, and spectral power in four frequency regions were computed using the Maximum Entropy Method (MEM): LF-band (0.01–0.05 Hz), MF1-band (0.05–0.10 Hz), MF2-band (0.10–0.15 Hz), and HF-band (0.15–0.20 Hz) according to Baria et al.^19,52^. A positive response in these bands is hypothesized to indicate how astronauts adapt to the space environment: the LF- and MF1-bands reflect an activation of the DMN’s medial prefrontal cortex (mPFC), posterior parietal cortex, posterior portion of precuneus and posterior cingulate cortex (PCC), while the MF2- and HF-bands show an activation of the orbitofrontal and temporal cortex parts of the DMN^52^.

The ISO (0.01–0.10 Hz) herein includes the LF-band (0.01–0.05 Hz), MF1-band (0.05–0.10 Hz) and VLF-component (0.003–0.04 Hz). We use the term “infraslow” herein because it is accepted in the field. Its semantics, however, is questionable. First, “infra”, derived from Latin, meaning “below” is juxtaposed with “slow”, an English term. Second, “infra” in “infrared” or in “infradian” correctly denotes wavelengths lower than those in the visible range, and frequencies lower than circadian, respectively. In “ultraslow”, however, “infra” is followed by the adjective “slow”, which conveys speed without providing a well-defined reference (slow as compared to what?). Since ISOs refer to oscillations with a period of about 1 min, they could be termed “infraSWS” (for “infraslow-wave-sleep”), a recommendation we hope will be considered by those in charge of the nomenclature.

### Cosine curve fitting for estimating amplitude and phase by cosinor

Single 24-h, 12-h, 8-h, or 3-h cosine curves were fitted independently to the data by cosinor^53–55^ to estimate their respective amplitudes and phases as well as rhythm-adjusted mean values (MESORs). Changes in biological rhythm amplitude assessed the response in rhythmicity of each biological rhythmic component to the space environment.

### Assessment of magnetic fluctuations

The ISS is protected from the space environment by Earth's magnetic field. The ISS orbits the Earth every 90 min at an altitude of 330 to 480 km. As previously reported^17,18^, to assess the effect of magnetic fluctuations on the ISS, we used geomagnetic measurements from the Auroral Observatory of the University of Tromsø, Norway (69°39’ N, 18°56’ E). We used 1-min data of total intensity (F, in nT), declination (D, angle between geographic and magnetic north, in degrees), inclination (I, angle between horizontal plane and magnetic direction, in degrees), horizontal intensity (H, in nT), and vertical intensity (Z, in nT).

Historically, the estimation of the ionospheric electric field used ionospheric currents and field-aligned currents from ground magnetic records, along with incoherent scatter radars, satellite measurements of X-ray and UV aurorae^56^. This type of study over the past two hundred years about the structure and temporal changes of the Sun–Earth space, now called space weather, has become of great interest to space science^57–61^.

### Statistical analyses

Data are expressed as mean ± standard deviation (SD). For comparison of HRV indices, statistical analyses are applied to hourly averages of the 5-min estimates. HRV endpoints during ISS01 and ISS02 are compared to before-flight (Before) by the paired t-test. The 3-, 8-, 12-, or 24-h amplitudes of HR and HRV endpoints are estimated by fitting single cosine curves to the 48-h data series by cosinor^53–55^. Indices of magnetic fluctuations are correlated to HRV endpoints in order to assess any effect of the space environment using Pearson’s correlation coefficient. The Stat Flex (Ver. 6) software (Artec Co., Ltd., Osaka, Japan) is used. P-values less than 0.05 are considered to indicate statistical significance.

## Results

### Adaptation of cardiovascular function during 1-year spaceflight

The astronaut’s disordered intrinsic cardiovascular regulatory system (β) improved on days 326–327 after launch, Table 1. After markedly declining during ISS01 from 0.942 ± 0.328 to 0.638 ± 0.257 (P < 0.01), it rebounded during ISS02 to 0.789 ± 0.305 (P < 0.05 vs. ISS01). This recovery of β on days 326–327 after launch is a new finding since our previous studies were limited to changes occurring during the first 6 months in space^4,5,17–19^. Accordingly, we examined which processes may have contributed to this improvement.

**Table 1:** Change in 48-h average of heart rate and heart rate variability during 12-month spaceflight ‡.

Variable (units)		ISO	Before (157–158 days before launch) (n = 44)	ISS01 (20–21 days after launch) (n = 46)		ISS02 (326–327 days after launch) (n = 44)			After (103–104 days after return to Earth) (n = 39)	
			Mean ± SD	Mean ± SD	ISS01/Before (%)	Mean ± SD	ISS02/Before (%)	ISS02/ISS01 (%)	Mean ± SD	After/Before (%)
**48-h average**										
Heart Rate	(beats/min)		70.8 ± 7.7	70.8 ± 6.6		70.9 ± 7.2			75.7 ± 6.3**	(106.9)
NN-intervals	msec		858.8 ± 88.6	855.4 ± 72.8		856.0 ± 69.9			799.9 ± 66.6**	(93.1)
r-MSSD	msec		16.1 ± 2.5	14.9 ± 1.6**	(92.5)	16.7 ± 2.2##		(112.1)	13.2 ± 2.4*	(82.0)
pNN50	%		0.59 ± 0.50	0.40 ± 0.32*	(67.8)	1.35 ± 0.71**##	(228.8)	(337.5)	0.50 ± 0.60	
TF	msec^2^		3579.8 ± 1263.4	3264.4 ± 1887.8		4378.1 ± 3841.0			3116.5 ± 1263.9	
ULF	msec^2^		1857.0 ± 1015.9	1373.5 ± 1614.3		2427.0 ± 3743.9			1602.2 ± 1005.5	
VLF	msec^2^	ISO	1322.2 ± 525.4	1350.8 ± 739.9		1320.6 ± 677.6			1085.5 ± 455.6*	(82.1)
LF	msec^2^		438.7 ± 171.9	464.7 ± 129.6		542.2 ± 163.8**##	(123.6)	(116.7)	379.7 ± 186.7	
LF/HF	–		12.9 ± 4.0	13.4 ± 4.0		13.0 ± 4.2			13.5 ± 3.9	
HF	msec^2^		35.8 ± 11.3	36.1 ± 7.7		44.0 ± 11.1**##	(122.9)	(121.9)	30.4 ± 12.2*	(84.9)
|β|	msec^2^/Hz		0.942 ± 0.328	0.638 ± 0.257**	(67.8)	0.789 ± 0.305*#	(83.7)	(123.5)	0.931 ± 0.343	
**Daytime average**										
Heart Rate	(beats/min)		73.2 ± 7.4	73.4 ± 5.9		73.3 ± 7.0			78.2 ± 5.3**	(106.8)
NN-intervals	msec		828.8 ± 77.8	823.1 ± 56.1		827.7 ± 58.3			772.1 ± 51.0**	(93.2)
r-MSSD	msec		16.5 ± 2.3	14.7 ± 1.6**	(89.3)	17.3 ± 2.1##		(117.6)	12.7 ± 2.3**	(77.1)
pNN50	%		0.54 ± 0.45	0.29 ± 0.18**	(53.2)	1.45 ± 0.75**##	(268.2)	(504.0)	0.41 ± 0.64	
TF	msec^2^		3903.9 ± 1346.6	3235.5 ± 2190.5		4804.7 ± 4469.5			3282.2 ± 1366.5	
ULF	msec^2^		2274.6 ± 980.5	1606.0 ± 1889.0		2954.7 ± 4331.5			1941.5 ± 962.2	
VLF	msec^2^	ISO	1235.3 ± 514.7	1123.1 ± 652.8		1209.6 ± 726.7			937.8 ± 386.4*	(75.9)
LF	msec^2^		454.5 ± 177.1	448.5 ± 96.5		538.6 ± 164.5##		(120.1)	368.7 ± 207.1	
LF/HF	–		13.0 ± 3.7	13.2 ± 3.1		11.5 ± 2.7#		(87.1)	13.6 ± 3.6	
HF	msec^2^		36.4 ± 9.7	35.7 ± 7.9		47.9 ± 9.8**##	(131.8)	(134.4)	27.7 ± 11.1**	(76.1)
|β|	msec^2^/Hz		1.080 ± 0.298	0.738 ± 0.228**	(68.3)	0.878 ± 0.300*#	(81.4)	(119.0)	1.080 ± 0.232	
**Night-time average**										
Heart Rate	(beats/min)		66.4 ± 6.4	64.1 ± 2.6		64.6 ± 2.2			69.1 ± 3.6	
NN-intervals	Msec		911.3 ± 83.6	937.3 ± 36.5		931.3 ± 31.9			870.7 ± 46.1	
r-MSSD	msec		15.6 ± 2.9	15.4 ± 1.5		15.3 ± 1.8			14.4 ± 2.3	
pNN50	%		0.66 ± 0.58	0.69 ± 0.40		1.08 ± 0.52#		(155.7)	0.73 ± 0.44	
TF	msec^2^		3032.7 ± 904.4	3328.7 ± 987.0		3311.5 ± 766.3			2740.0 ± 940.8	
ULF	msec^2^		1152.4 ± 616.3	854.7 ± 394.4		1107.8 ± 349.2			831.2 ± 612.4	
VLF	msec^2^	ISO	1474.3 ± 525.0	1928.8 ± 642.1*	(130.8)	1616.5 ± 418.6			1461.5 ± 411.1	
LF	msec^2^		411.1 ± 164.1	505.9 ± 188.7		551.8 ± 168.6*	(134.2)		407.8 ± 124.1	
LF/HF	–		12.6 ± 4.7	14.0 ± 5.8		17.0 ± 5.0*	(135.6)		13.3 ± 4.8	
HF	msec^2^		34.8 ± 14.0	37.4 ± 7.5		33.5 ± 6.9			37.3 ± 12.6	
|β|	msec^2^/Hz		0.702 ± 0.225	0.416 ± 0.164**	(59.4)	0.564 ± 0.181#		(135.4)	0.549 ± 0.282	

ISO: Infraslow Oscillation (0.01–0.10 Hz).

TF: (0.0001–0.5) Hz; ULF: (0.0001–0.003) Hz; VLF: (0.003–0.04) Hz; LF: (0.04–0.15) Hz; HF: (0.15–0.40) Hz; β: (0.00013–0.02) Hz.

|β|: slope of fractal scaling, corresponding to periods of 120 to 0.83 min, reflecting the intrinsic autonomic regulatory system.

^‡^Tests applied to hourly averages of 5-min intervals in order to eliminate or at least reduce serial correlation.

*P < 0.05; **P < 0.01: ISS01, ISS02 or After vs.Before ; #P < 0.05; ##P < 0.01: ISS02 vs. ISS01.

P-values from Student t-test; SD = standard deviation.

n: number of hourly averages (of 5-min intervals).

First, on days 20–21 after launch (ISS01), when β decreased, the parasympathetic activity was suppressed. This is reflected by changes in r-MSSD and pNN50, which dropped to 92.5% (P < 0.01) and 67.8% (P < 0.05) of pre-launch values, respectively, Table 1 (top). On days 326–327 after launch (ISS02), when β had rebounded, both r-MSSD (P < 0.01 vs. ISS01) and pNN50 (P < 0.01 vs. ISS01) increased. In addition, LF (P < 0.01 vs. pre-launch and vs. ISS01) and HF (P < 0.01 vs. pre-launch and vs. ISS01) also increased. It thus seems that suppression or recovery of parasympathetic activity participated in changes observed for β. When considering daytime and nighttime changes separately, already during ISS01, when the parasympathetic activity was suppressed, the VLF component increased, but only during the night (P < 0.05 vs. pre-launch). This result suggests that an activation of the ISO first started at night, Table 1 (bottom).

### Changes in circadian and ultradian amplitudes of heart rate variability during spaceflight

Second, increases in the circadian amplitudes of HR and HRV suggest that the circadian system is amplified during spaceflight. Already during ISS01, when β was decreased, the circadian amplitude of HR increased by 36.6%, from 4.8 (Before) to 6.6 bpm, as did the circadian amplitude of VLF (by 10.7%), which represents the ISO. During ISS02, when β rebounded, the circadian variation of HR remained increased, Table 2 (top), and the circadian amplitude of other HRV endpoints also gained in prominence. The circadian amplitude of TF increased by 108.4%, that of ULF, which fluctuates more slowly than ISOs, increased by 108.7%, and that of HF, which reflects parasympathetic activity, increased by 113.1%.

Increases in the variability of TF and ULF are already observed during ISS01 in terms of the 12-h (circasemidian, by 94.5% and 105.8%, respectively) and 8-h (by 86.9% and 279.6%, respectively) amplitudes, Table 2 (middle). An increased 3-h amplitude was observed not only for TF (169.1%), but also for NN-intervals (548.6%), VLF (128.5%), LF (178.2%), and HF (103.6%), Table 2 (bottom).

### Activation of default mode network starts at night

Third, we examined responses of four HRV endpoints in every frequency band from 0.01 to 0.20 Hz, during the entire 48-h record as well as during daytime and nighttime separately, Table 3. During ISS01, statistically significant increases were found during sleep (night) in the spectral power of the LF-band (p < 0.01) and MF1-band (P < 0.05) as compared to pre-launch. During ISS02, the adaptation response changed: while a positive response of the LF-band (0.01–0.05 Hz) at night was no longer found, the spectral power of the MF1-band (0.05–0.10 Hz) remained increased compared to pre-launch (P < 0.01), Table 3. These results, together with the VLF response (30.8% increase at night, P < 0.05, Table 1), suggest that changes in brain activity (in brain’s functional neural networks) first started at night. Whole-day analysis during ISS02 shows a positive response of the MF1-band (P < 0.01) associated with amplification of the MF2- and HF-bands (P < 0.01 both), Table 3. These changes reflect the activation of the brain’s functional connectivity from the DMN to the alerted DMN, as discussed previously^19^.

**Table 2:** Changes in amplitudes of heart rate and heart rate variability during 12-month spaceflight.

	Variable and (units) or frequency range (Hz)		Before (estimate)	ISS01 (estimate)	ISS01/Before (%)	ISS02 (estimate)	ISS02/ Before (%)	After (estimate)	After/Before (%)
Circadian (24-hour) amplitude	HR	(beats/min)	4.8	6.6	(136.6)	6.5	(134.4)	5	(102.5)
	NN-intervals	(msec)	59.7	76.8	(128.6)	71.5	(119.7)	52.9	(88.5)
	TF	0.0001-0.5	902.9	631.4	(69.9)	**1881.4**	**(208.4)**	873.8	(96.8)
	ULF	0.0001-0.003	896.3	864.4	(96.4)	**1870.7**	**(208.7)**	588.6	(65.7)
	VLF	0.003-0.04	395.4	437.7	(110.7)	334.7	(84.6)	322.5	(81.6)
	LF	0.04-0.15	73.2	12.9	(17.7)	47.8	(65.3)	**115.8**	**(158.2)**
	HF	0.15-0.40	5.42	6.23	(114.9)	**11.6**	**(213.1)**	**8.73**	**(161.1)**
Circasemidian (12-hour) amplitude	HR	(beats/min)	2.9	2.1	(73.7)	1.3	(46.2)	3.1	(109.2)
	NN-intervals	(msec)	30.7	28.4	(92.5)	18.3	(59.6)	33.6	(109.4)
	TF	0.0001-0.5	664.1	**1291.8**	**(194.5)**	**1819.9**	**(274.0)**	703.1	(105.9)
	ULF	0.0001-0.003	415.5	**855.1**	**(205.8)**	**1492.9**	**(359.3)**	**697.1**	**(167.8)**
	VLF	0.003-0.04	307.0	452.0	(147.2)	389.9	(127.0)	251.3	(81.9)
	LF	0.04-0.15	71.5	25.6	(35.8)	59.4	(83.1)	41.9	(58.7)
	HF	0.15-0.40	3.72	1.52	(41.0)	2.73	(73.4)	**6.27**	**(168.6)**
Ultradian (8-hour) amplitude	HR	(beats/min)	1.6	2.2	(135.0)	2.0	(122.2)	0.1	(3.3)
	NN-intervals	(msec)	19.8	23.9	(120.4)	20.7	(104.3)	3.4	(16.9)
	TF	0.0001-0.5	335.5	**627.0**	**(186.9)**	355.8	(106.1)	240.3	(71.6)
	ULF	0.0001-0.003	166.6	**632.4**	**(379.6)**	182.5	(109.5)	121.1	(72.7)
	VLF	0.003-0.04	187.8	50.4	(26.8)	189.2	(100.8)	49.4	(26.3)
	LF	0.04-0.15	79.5	43.0	(54.1)	61.9	(77.9)	59.3	(74.6)
	HF	0.15-0.40	1.81	**2.92**	**(161.5)**	**3.62**	**(200.1)**	0.97	(53.7)
Ultradian (3-hour) amplitude	HR	(beats/min)	0.3	**1.2**	**(388.7)**	**1.0**	**(326.6)**	0.4	(138.3)
	NN-intervals	(msec)	1.7	**11.0**	**(648.6)**	**7.5**	**(445.5)**	**5.4**	**(317.5)**
	TF	0.0001-0.5	92.2	**248.0**	**(269.1)**	**606.7**	**(658.3)**	72.3	(78.5)
	ULF	0.0001-0.003	80.9	60.1	(74.2)	**550.9**	**(680.7)**	9.3	(11.4)
	VLF	0.003-0.04	126.1	**288.2**	**(228.5)**	137.1	(108.7)	62.0	(49.2)
	LF	0.04-0.15	24.2	**67.4**	**(278.2)**	17.5	(72.3)	19.6	(81.0)
	HF	0.15-0.40	0.58	**1.18**	**(203.6)**	**1.28**	**(220.7)**	0.51	(88.1)

Bold areas show increases by more than 150% compared to Before.

**Table 3:** Changes in heart rate variability reflecting the Default Mode Network as a function of time spent in space ‡.

Variable (frequency range, Hz)		ISO	Before (157–158 days before launch)		ISS01 (20–21 days after launch)			ISS02 (326–327 days after launch)				After (103–104 days after return to Earth)		
		(0.01–0.10 Hz)	n	Mean ± SD (msec^2^)	n	Mean ± SD (msec^2^)	ISS01/Before (%)	n	Mean ± SD (msec^2^)	ISS02/Before (%)	ISS02/ISS01 (%)	n	Mean ± SD (msec^2^)	After/Before (%)
**48-h (whole day)**														
LF-band	0.01–0.05	ISO	44	779.2 ± 368.5	46	923.1 ± 472.8		44	861.4 ± 418.2			39	650.9 ± 340.9	
MF1-band	0.05–0.10	ISO	44	290.0 ± 124.0	46	316.7 ± 80.7		44	365.1 ± 111.1**#	(125.9)	(115.3)	39	256.4 ± 125.6	
MF2-band	0.10–0.15		44	53.3 ± 21.6	46	53.4 ± 17.2		44	76.7 ± 33.8**##	(143.9)	(143.6)	39	48.4 ± 24.5	
HF-band	0.15–0.20		44	12.2 ± 4.5	46	12.8 ± 4.1		44	16.7 ± 7.0**##	(136.9)	(130.5)	39	10.3 ± 4.8	
**Daytime (awake span)**														
LF-band	0.01–0.05	ISO	28	658.9 ± 338.0	33	728.8 ± 307.6		32	754.1 ± 400.4			28	509.6 ± 225.7	
MF1-band	0.05–0.10	ISO	28	312.2 ± 133.7	33	310.8 ± 67.9		32	363.2 ± 112.2#		(116.9)	28	250.8 ± 141.6	
MF2-band	0.10–0.15		28	59.8 ± 21.2	33	59.5 ± 15.0		32	86.9 ± 32.8**##	(145.3)	(146.1)	28	53.0 ± 27.4	
HF-band	0.15–0.20		28	13.6 ± 4.7	33	14.5 ± 3.3		32	19.5 ± 6.0**##	(143.3)	(134.5)	28	11.2 ± 5.3	
**Nighttime (sleep span)**														
LF-band	0.01–0.05	ISO	16	989.8 ± 330.6	13	1416.4 ± 467.5**	(143.1)	12	1147.6 ± 330.0			11	1010.7 ± 325.0	
MF1-band	0.05–0.10	ISO	16	251.1 ± 96.9	13	331.4 ± 108.7*	(132.0)	12	370.1 ± 112.5**	(147.4)		11	270.8 ± 74.3	
MF2-band	0.10–0.15		16	41.8 ± 17.6	13	38.0 ± 12.3		12	49.5 ± 18.5			11	36.8 ± 7.3	
HF-band	0.15–0.20		16	9.9 ± 3.2	13	8.6 ± 2.7		12	9.3 ± 2.7			11	8.1 ± 1.6	

ISO: Infraslow Oscillation.

^‡^Tests applied on hourly averages of 5-min intervals in order to eliminate or at least reduce serial correlation.

*P < 0.05; **P < 0.01: ISS01, ISS02 or After vs. Before ; #P < 0.05; ##P < 0.01: ISS02 vs. ISS01 P-values from Student t-test; SD = standard deviation.

|β|: slope of fractal scaling, corresponding to periods of 120 to 0.83 min, reflecting the intrinsic autonomic regulatory system.

n: number of hourly averages (of 5-min intervals).

### Effects of magnetic fluctuations on the ISOs in space

**Figure 1:**
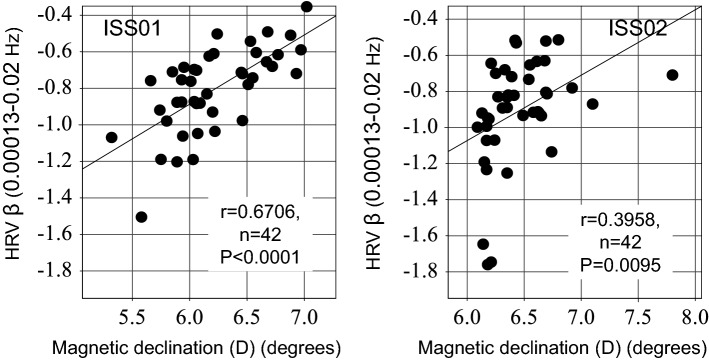
Effects of magnetic fluctuations on the “intrinsic” cardiovascular regulatory system (β). Magnetic declination (D) correlates positively and statistically significantly with the “intrinsic” cardiovascular regulatory system (β) during both ISS01 (r = 0.6706, P < 0.0001) and ISS02 (r = 0.3958, *P* = 0.0095). These results suggest that the improvement in β may relate to magnetic fluctuations in space, and that the effect may last during the entire 12-month spaceflight. Pearson’s correlation coefficients computed from hourly averages of magnetic declination (D) and HRV β in order to eliminate or at least reduce serial correlation.

The fourth probable factor contributing to the rebound of β may involve effects of magnetic fluctuations on HRV endpoints. Among geomagnetic indices, the geomagnetic declination (D) showed the strongest association. We found the same result in an earlier study on 10 other healthy astronauts spending 6 months on the ISS^18^. As shown in Fig. 1, D correlates positively with the “intrinsic” cardiovascular regulatory system (β) during both ISS01 (r = 0.6706, P < 0.0001) and ISS02 (r = 0.3958, *P* = 0.0095). These results suggest that the rebound of β may depend on the state of changes in the magnetic field in space and that these effects may be long-lasting.

**Figure 2:**
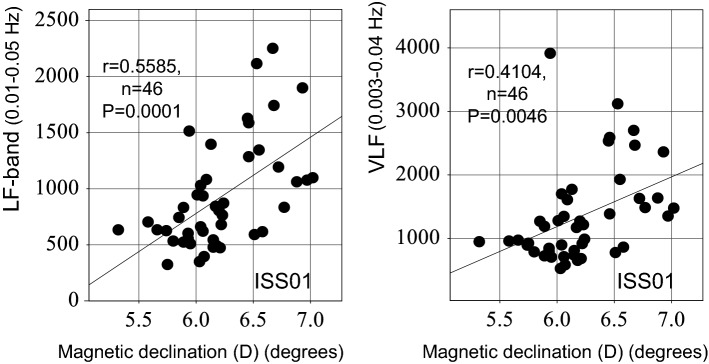
Effects of magnetic fluctuations on the ISO, LF-band and VLF-component. Magnetic declination (D) positively affects the LF-band (r = 0.5585, *P* = 0.0001) (left) and the VLF-component (r = 0.4104, *P* = 0.0046) (right) during ISS01, suggesting that the activation of the brain’s DMN by magnetic fluctuations may play an important role for improving the “intrinsic” cardiovascular regulatory system (β) in space. Pearson’s correlation coefficients computed from hourly averages of magnetic declination (D) and ISOs (LF-band and VLF component of HRV) in order to eliminate or at least reduce serial correlation.

Magnetic fluctuations positively affected the LF-band (r = 0.5585, *P* = 0.0001) during ISS01, Fig. 2 (left), suggesting that the activation of the brain’s DMN by magnetic fluctuations may have played an important role in improving the “intrinsic” cardiovascular regulatory system (β) in space. Magnetic fluctuations also affected positively VLF (r = 0.4104, *P* = 0.0046) during ISS01, Fig. 2 (right), suggesting that the activation of the ISO by magnetic fluctuations may have played a significant role in improving β in space.

### Incidence of circadian desynchrony after spaceflight

**Figure 3:**
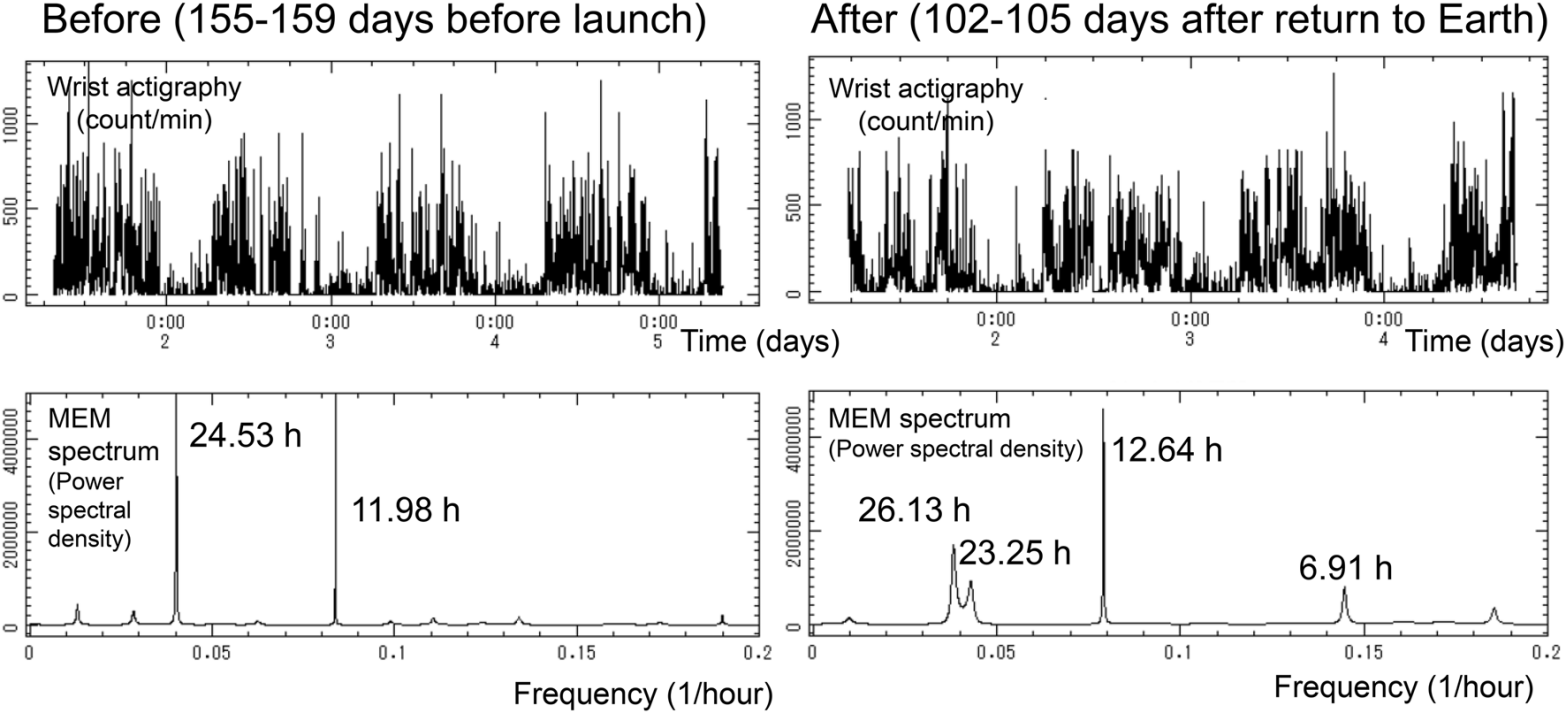
MEM spectral analysis of actigraphy records spanning about 84 h before (left) and after (right) the mission in space. Left: Circadian rhythm with an estimated period of 24.53 h is detected together with a circasemidian component with an estimated period of 11.98 h. Right: Decreased circadian prominence along with split of circadian rhythm into two components with estimated periods of 26.13 and 23.25 h, suggesting the occurrence of social jetlag after return to Earth.

Upon return to Earth, the organism needs to adapt from space microgravity to gravity on Earth. HR was slightly higher compared to pre-launch (P < 0.05). Parasympathetic activity (reflected by r-MSSD and HF) decreased compared to ISS02 and were even lower than pre-launch (P < 0.05 both). VLF (one of the ISOs) was also lower compared to pre-launch P < 0.05), Table 1 (top). These results are likely affected by social jetlag after return to Earth, when the circadian amplitude of activity is reduced and the circadian rhythm may be split into two components with periods of 26.13 and 23.25 h, as shown in actigraphy data recorded over about 84 h, Fig. 3.

## Discussion

Spaceflight dramatically alters the intrinsic cardiovascular regulatory system. As reported earlier^4,5,17–19^, it did not adapt to microgravity after 6 months in space. The monitoring of a healthy astronaut who spent 12 months in space gave us a unique opportunity to examine the adaptive process of the neural cardiovascular coordination beyond the previous missions lasting only 6 months. As NASA started a 1-year mission project^12,13^ and more long-term missions are planned, learning how to facilitate human adaptation to space microgravity is urgently needed. We discovered that the alteration in the intrinsic cardiovascular regulatory system (β) observed after 1 month in space was significantly less 10 months later (*P* = 0.0167), although the rebound was incomplete, Table 1.

### Role of biological rhythms in the adaptation to the space environment

Whereas the circadian system plays a key role in the adaptation to the novel space environment^4,5,17–19^, new evidence suggests that ultradian components provided an evolutionary advantage for almost all life forms, from bacteria to humans^62–64^. These ultradian rhythms can be expected to be important for the rapid adaptation to microgravity in space.

In addition to a robust amplification of the circadian variation of HR, TF, ULF, and HF, we observed a larger than twofold increase in the amplitude of the 12-h (circasemidian), 8-h and even 3-h oscillations of HRV indices, Table 2. The ultradian response appeared faster and was larger than the circadian response, even during ISS01. The 12-h component may reflect the function of the “endogenous endoplasmic reticulum (ER) stress and unfolded protein response (UPR) cycle”^64–68^, while the amplified 8-h oscillations may indicate the involvement of the immune function by means of NF-kB signaling^62^, and the intensified 3-hourly variations suggest the participation of the hypothalamic–pituitary–adrenal (HPA) axis^69–71^. These observations show that when faced to a new environment, humans begin by adapting to ER stress in order to survive, and later enhance their immune and HPA axis functions. Harmonic oscillations of 24, 12, 8 and 3 h likely provide evolutionarily adaptive advantages. The contribution of ultradian components to consolidating a strong circadian system in space may contribute to an anti-aging effect noted in other investigations^10–12,16–18^.

### Activation of the DMN outside of consciousness

The brain’s DMN may serve a broader adaptive purpose, in response to uncertainty, threats, and novel environments^52,72–77^. Only the DMN functional connectivity was reported to change post-flight^78^. Baria et al.^52^ examined spatial organizational rules for the human brain oscillatory activity as measured by the blood oxygen level-dependent (BOLD) signal. BOLD oscillation properties of the DMN were shown to consist of distinct frequency-dependent regions: the LF-band reportedly localized mainly to the *prefrontal*, *parietal*, and *occipital cortex*; the MF1-band to the *thalamus* and *basal ganglia*; the MF2-band to the *orbitofrontal cortex*, *temporal cortex* and *insula*; and the HF-band to the *insula*, *temporal cortex* and *subcortical regions*^52^.

HRV may serve as a useful marker since it varies in concert with changes in brain functional connectivity. We previously investigated how the DMN may facilitate adaptation to space by assessing HRV changes in 8 astronauts examined repeatedly during their 6-month space missions^17^. We here used the same four frequency-band HRV classification as Baria et al.^52^ to analyze DMN’s role in the adaptation to space. We find that the rebound in β was associated with the activation of the DMN at night, gauged by the 43.1% increased spectral power in the LF-band (*P* = 0.0078), which was later extended to the MF1-band observed over the entire 24-h day but remaining larger at night than during the day, Table 2. These results suggest that an activation of the adaptive process by the DMN starts at night and spreads to the entire day as time goes by.

After one year in space, spectral power in the MF1-, MF2- and HF-bands increased over the entire 24-h day (also during daytime), albeit without any further increase in the LF-band. A recovery process of disordered cardiovascular dynamics thus occurred in this case over the span of one year in space. It was likely mediated by activating the DMN in a circadian stage-dependent manner, without conscious awareness. The circadian stage-dependent activation of the DMN may help restore human neural cardiovascular coordination in space, perhaps contributing to an anti-aging effect.

### Nighttime and role of SCN astrocytes

The adaptive process of disordered cardiovascular dynamics was found to be circadian stage-dependent, starting at night and later spreading to the entire day. These observations are consistent with recent studies showing that SCN neurons and also SCN astrocytes coordinate overt circadian behavior, illustrating the intrinsically dual nature of the clock circuit in mammals^27,28^. Astrocytes were thought to play a supporting role for neurons, by providing energy and other nutrients, repairing the nervous system, and clearing ions and neurotransmitters during neurotransmission^26,79^. Astrocytes have been demonstrated, however, to have robust rhythms in their activity, as evidenced by intracellular calcium^27^, and to be part of the circadian circuit coordinating circadian behavior.

Astrocytes are cells characterized by circadian rhythms, just as neurons are. The circadian rhythm of neuronal activity peaks during the daytime, around ZT06, whereas that of astrocytes activity peaks during the night, around ZT18, in antiphase with SCN neurons. Surprisingly, the SCN circadian network incorporates two functionally distinct cellular populations: day-active neurons and night-active astrocytes harnessing a differentially phased Transcription-Translation Feedback Loop (TTFL)^80^. During the night, SCN astrocytes inhibit SCN neurons by releasing gliotransmitters into the extracellular space, which may provide an inhibitory astrocytic-neuronal coupling signal^81^.

Several reasons exist why adaptation of cardiovascular dynamics to space unconsciously starts at night. Astrocytic networks are able to fundamentally control neuronal network activity, and to significantly contribute to the generation (or spreading) of oscillations across the SCN to broad brain relevance^82^. The astrocytic network should thus be a key player in brain oscillations. SCN astrocytes are also capable of timekeeping under constant darkness, or in constant light^83^. Moreover, not only are the astrocytes embedded in the clock circuit, astrocyte clocks contribute to the coordination of period length in SCN function^80,84^. Astrocytes are thus important for the adaptation of free-running rhythms in space and for their synchronization to the artificial 24-h day on the ISS during long-duration spaceflights. In view of the critical importance of synchronized circadian rhythms in relation to health, these results may help explain their contribution to an anti-aging effect. Our observations herein concur with the idea that SCN astrocytes also play an important role for biological timekeeping alongside SCN neurons (“Move over neurobiology — astrobiology is dawning”)^85^.

### ISOs and role of thalamic astrocytes

Since the early 2000s, the ISO, defined as a brain electrical rhythm with maximal spectral power centered around 0.02 Hz (about 1/min), has received increased attention because it is considered to reflect fluctuations underlying brain functional networks in co-varying brain regions. ISOs interact with a variety of neural functions to affect both sleep dynamics and cognition during wake states. ISOs are even thought to play a coordinating role in brain dynamics.

The mechanism of this oscillation was found in the thalamus and may ultimately come from glial cells^86^. Thalamic astrocytes may be key to the mechanism of the ISO^87,88^. In fMRI temporal signals, ISOs have been found during sleep^89–91^. Only when ISOs dominate the power spectrogram of human EEG signals during sleep, deep slow-wave sleep is present, and a deeper level of slow-wave sleep appears with an increase in ISO power^92^. Extensive synchronization of the astrocytic network advances neural coupling in slow-wave sleep^93^.

The LF-band (0.01–0.05 Hz), MF1-band (0.05–0.10 Hz), or VLF component (0.003–0.04 Hz) with a peak at 0.02 Hz correspond to the ISOs. As shown in Table 1, VLF measures only showed any changes in response to the space environment during the night. It was then already increased during ISS01 (1928.8 ± 642.1 msec^2^) as compared to pre-launch (1474.3 ± 525.0 msec^2^) and remained higher during ISS02 (1616.5 ± 418.6 msec^2^) before returning to pre-launch values after return to Earth (1461.5 ± 411.1 msec^2^). Adaptation to the space environment may thus have started at night, mediated by an increase in ISO power, which reflects highly thalamic-dependent astrocytes, notably during sleep.

As glial, mainly astrocytic, networks may underlie the ISO, the effect of rhythmic glial events upon thalamic neurons may have a widespread influence outside of consciousness in a variety of brain functional networks. It can be expected that the ISO will provide more information regarding effects of living in space, notably about how and when humans can adapt to it^20–23,94,95^. ISOs and their role in promoting deep slow-wave sleep may have contributed to any anti-aging effect of the space environment.

### Magnetic fluctuations and the ISO

In a series of studies, Breus et al.^96–100^ proposed that variations in the earth's magnetic field and magnetic storms present a risk for the development of cardiovascular disorders. These authors also investigated how geomagnetic storms influence crewmembers during space missions onboard Soyuz, Mir and ISS^101,102^. They reported HRV responses that differed depending on the state of adaptation. After 1 month in space, geomagnetic storms increased HR and decreased r-MSSD and pNN50, whereas after 6 months in space, HR decreased while r-MSSD and pNN50 increased^102^.

The present study shows for the first time how geomagnetic fluctuations influence the “intrinsic” cardiovascular regulatory system (β) and ISOs in space, apart from effects on conventional HRV indices. Specifically, we found relations between magnetic fluctuations and the cardiovascular regulatory system β (Fig. 1) and ISOs (LF-band and VLF component of HRV), Fig. 2. During ISS01, the magnetic declination correlated positively with β (r = 0.6706, P < 0.0001), LF-band (r = 0.5585, *P* = 0.0001), and VLF (r = 0.4104, *P* = 0.0046), suggesting that magnetic fluctuations may affect ISOs and improve the disturbed cardiovascular regulatory system. During ISS02, however, a correlation with ISOs (LF-band and VLF) was no longer significant, but the association persisted with the cardiovascular regulatory system β. The influence of magnetic fluctuations on β depending on the ISO was limited to the early days on the ISS. Whereas the study is limited to a single astronaut, the results extend those obtained earlier during shorter missions. Adaptation of human neural cardiovascular coordination to the space environment remains a challenge, as mechanisms are diverse and complex.

### Brain astrocytes and adaptation to space mediated by conscious mental processes

After 11 months in space, an increase in MF1-band power was observed not only at night (during sleep), but also during the day (while awake), Table 3. As discussed previously^19^, adaptation to space microgravity proceeded mainly by the unconscious mind. The MF1-band response observed in this investigation, however, also occurred during the day, in full consciousness.

Most brain cells are not neurons but glial cells (astrocytes, oligodendrocytes, and microglia). Increasing evidence suggests that glial cells play an important role in various brain functions. The interaction between neurons and glia may clarify unknown aspects, including conscious-unconscious relationships^103^. Consciousness is self-awareness, which is vital when solving explicit tasks. Astrocyte gap junctions have recently been proposed to be the locus of conscious representation, postulated to occur when gap junctions are activated by synaptic activity to form a three-dimensional global network^26,91,104^.

### Social jetlag after return to Earth

After return to Earth, HR was higher as compared to pre-launch. Parasympathetic activity, expressed by r-MSSD or HF, and VLF decreased, Table 1. MEM spectral analysis of about 84-h actigraphy records obtained before and after the space mission shows that the originally 24-h synchronized circadian rhythm was weaker and split into two near-24-h components after return to Earth, Fig. 3, suggesting the occurrence of social jetlag related to busy work schedules on Earth.

### Limitations

This investigation has several limitations. Limited to a single astronaut, the study is also limited by the fact that geomagnetic measures in Tromsø, Norway, were used instead of space weather measurements on the ISS. Relatively short ECG records of only 48 h were obtained only twice during a 12-month space mission. Longer, continuous ECG records are desirable to clarify actual fluctuations in adaptation processes in space, as suggested previously^55,105,106^. While brain oscillatory activity data are lacking, studies have shown that HRV is associated with structures and functions of the neural network, and that HRV is a biomarker reflecting activities of the brain integration system. Since these associations are complex, they need to be confirmed in future investigations to directly depict brain oscillatory activity in space.

### Conclusion

The intrinsic cardiovascular regulatory system (β) shows unequivocal changes in space in our previous investigations^4,5,17–19^ and it never adapted to microgravity after a 6-month spaceflight. The present investigation shows that the change of β at 1-month after launch improved after 11 months in space. This result is impressive since adjustment of the autonomic regulatory system will be critical for the correct physiological functioning in space as plans consider inhabiting Moon or Mars. Although results herein stem from only one astronaut, they support the idea that HRV provides insight into the degree to which adaptation to the new space environment is integrated into the brain’s default functional connectivity in association with SCN astrocytes and/or thalamic astrocytes. The HRV LF-, MF1- and MF2-bands, as well as HRV VLF may be especially important endpoints probing organismic functions associated with adaptability and health. Consolidation of a synchronized and amplified circadian system, together with ISO’s role in promoting deep slow-wave sleep may have contributed to anti-aging effects of the space environment noted in several investigations^10–12,16–18^. The next task will be to investigate more precisely the relationship between the level of consciousness (sleep versus awake states; passive versus active states) and DMN activity in terms of adaptive functions in space. Such studies could determine whether mechanisms of adaptation to weightless conditions are associated with activation of higher autonomic centers, as proposed by Baevsky et al.^30,44,45^. Consideration of ultradian components, together with ISOs of the vital brain–heart activity into medicine is an enormous challenge that may shape the future of science and offer an unprecedented opportunity for human advancement.

## Data Availability

Restrictions from Japan’s Aerospace Exploration Agency apply to the availability of the data supporting the findings of this study, which were used under license for the current study. As such, they are not publicly available.
